# Natural Product 2-Oxokolavenol Is a Novel FXR Agonist

**DOI:** 10.3390/molecules27248968

**Published:** 2022-12-16

**Authors:** Fusheng Guo, Yihui Gao, Xiaobao Li, Xiaoguang Lei

**Affiliations:** 1 Beijing National Laboratory for Molecular Sciences, Key Laboratory of Bioorganic Chemistry and Molecular Engineering of Ministry of Education, Department of Chemical Biology, College of Chemistry and Molecular Engineering, Synthetic and Functional Biomolecules Center, Peking University, Beijing 100871, China; 2 Peking-Tsinghua Center for Life Science, Academy for Advanced Interdisciplinary Studies, Peking University, Beijing 100871, China; 3 Key Laboratory of Tropical Medicinal Resource Chemistry of Ministry of Education & Key Laboratory of Tropical Medicinal Plant Chemistry of Hainan Province, College of Chemistry and Chemical Engineering, Hainan Normal University, Haikou 571158, China

**Keywords:** high-throughput screening, natural product, 2-oxokolavenol, FXR, mode of action

## Abstract

Acetaminophen (APAP) toxicity is a common cause of hepatic failure, and the development of effective therapy is still urgently needed. Farnesoid X receptor (FXR), a member of the nuclear receptor superfamily, has been identified as a master gene for regulating enterohepatic metabolic homeostasis and has proven to be a promising drug target for various liver diseases. Through high-throughput chemical screening, the natural product 2-oxokolavenol was identified as a novel and selective FXR agonist. Further investigations revealed that 2-oxokolavenol exerts therapeutic efficacy against APAP-induced hepatocyte damage in an FXR-dependent manner. Mechanistically, 2-oxokolavenol forms two hydrogen bonds with M265 and Y369 of human FXR to compatibly fit into the ligand binding pocket of FXR, which potently leads to the recruitment of multiple co-regulators and selectively induces the transcriptional activity of FXR. Our findings thus not only reveal the direct target of natural product 2-oxokolavenol, but also provide a promising hit compound for the design of new FXR modulators with potential clinical value.

## 1. Introduction

Acetaminophen (APAP) is one of the most commonly chosen drugs with analgesic and antipyretic properties [1,2]. In the United States, approximately 50 million individuals consume APAP in various forms every week, and APAP overdose and misuse have been the leading cause of acute liver failure worldwide [3,4]. However, the mechanism of APAP overdose-caused hepatic disorders remains complex and poorly understood. Accordingly, the existing therapies are not very effective. 

Farnesoid X receptor (FXR), a member of the nuclear receptor superfamily that is mainly expressed in enterohepatic tissues, has been discovered as a crucial regulator for balancing liver inflammatory processes and metabolism [5,6,7] and thus has become a pharmacological target for liver disorders. Bile acids were identified as endogenous ligands of FXR, but they also activate receptors other than FXR, indicating their poor selectivity, and many derivatives have been generated from the chemical modification of bile acids to gain more potent and selective FXR agonists [8,9]. Obeticholic acid (OCA), a semisynthetic bile acid derivate and full agonist of FXR, has been approved for the treatment of primary biliary cirrhosis (PBC) [10] and has also been evaluated in a phase III clinical trial for nonalcoholic steatohepatitis (NASH) treatment [11,12]. In addition, numerous FXR modulators have been discovered and their efficacy has been evaluated for the treatment of FXR-mediated liver diseases, such as PBC, NASH, biliary atresia, and APAP-induced liver failure. GW4064, the first synthetic FXR agonist, enhanced hepatocyte proliferation and alleviated APAP-induced cellular toxicity by activating FXR [13]. Hedragonic acid, a reported FXR agonist, protected the liver of mice from acute damage caused by excess APAP and reversed the inflammatory response in an FXR-dependent manner [14]. Cafestol, a dual agonist of FXR and PXR, has a potential role in cholesterol homeostasis [15]. Fexaramine is an intestine-restricted FXR agonist with limited activity targeting FXR in the liver [16,17]. WAY-362450, a potent and selective FXR agonist, alleviated NASH lesions by reversing liver inflammation and fibrosis in vivo [5,18]. The antiparasitic drugs ivermectin, doramectin, and abamectin also achieved their efficacy in regulating metabolism syndrome, including NAFLD, by directly targeting FXR [19,20]. Encouragingly, an increasing number of FXR modulators have already entered clinical trials, including EDP-305 [21], MET409 [22], TERN-101 [23], cilofexor [24,25], and tropifexor [26,27]. However, OCA is the only FDA-approved therapy in a clinical setting that directly targets FXR, and several side effects, such as severe pruritus and raised LDL levels, have severely hampered clinical applications in the future [11,28,29]. Therefore, given the importance of FXR in regulating enterohepatic metabolism and the inflammatory response, as well as the scarcity of efficient FXR modulators in the clinic, the discovery of novel FXR modulators and investigating their mode of action will have both scientific significance and therapeutic potential.

Natural products contain a wide range of biological activities and have proven to be a rich source for drug discovery [30,31]. 2-Oxokolavenol is a natural product isolated from the twigs of *Amoora stellato-squamosa* without any biological targets identified to date [32]. Here, through high-throughput screening and subsequent biological evaluation, this study identifies 2-oxokolavenol as a novel FXR agonist with therapeutic potential in treating APAP-induced hepatocyte damage, providing a promising hit compound for the future development of FXR-selective modulators with therapeutic potential in treating liver diseases.

## 2. Results

### 2.1. Identification of the Natural Product 2-Oxokolavenol as a Novel FXR Agonist 

**Figure 1:**
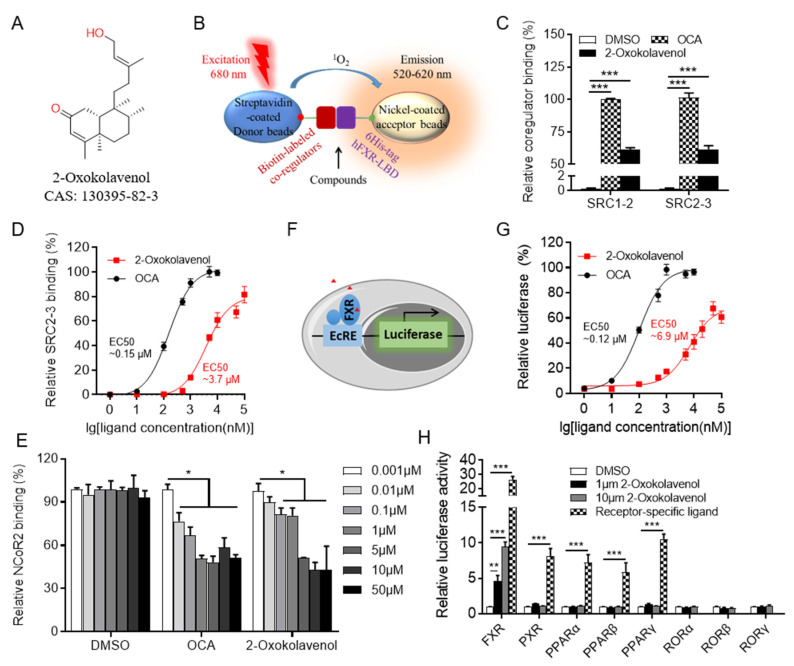
Identification of natural product 2-oxokolavenol as a novel FXR agonist. (**A**) Chemical structure of 2-oxokolavenol. (**B**) Diagram of the AlphaScreen assay for determining whether the tested compounds can induce FXR to recruit co-regulators. Biotin-labeled co-regulators bound to the streptavidin-coated donor beads, 6His-tagged FXR-LBD bound to the nickel-coated acceptor beads. (**C**) SRC1-2 and SRC2-3 coactivator motifs bound to FXR-LBD in response to 2-oxokolavenol or OCA in the AlphaScreen assay. (**D**) Dose response curves of 2-oxokolavenol or OCA in inducing FXR-LBD to recruit the SRC2-3 binding motif in the AlphaScreen assay. (**E**) NCoR2 corepressor motif bound to FXR-LBD in response to gradient 2-oxokolavenol or OCA in the AlphaScreen assay. (**F**) Diagram of cell-based luciferase reporter assay. FXR can bind to the ecdysone response element (EcRE) and initiate the transcription of downstream genes. Cloning the luciferase gene downstream of EcRE to gain the reporter assay. (**G**) Dose response curves of 2-oxokolavenol or OCA in inducing FXR transcriptional activity in the dual-luciferase reporter assay. (**H**) Selective transcriptional activity of FXR by 2-oxokolavenol based on dual-luciferase reporter assay. Plasmids encoding indicated full-length nuclear receptors and their cognate response reporters were co-transfected into HEK293T (from ATCC). Six hours later, cells were treated with DMSO, 2-oxokolavenol (1 μM and 10 μM), or receptor-specific agonist: FXR, 1 μM OCA; pregnane X receptor (PXR), 10 μM rifampicin; peroxisome proliferator-activated receptor alpha (PPARα), 1 μM GW590735; PPARβ, 1 μM GW0742; PPARγ, 1 μM rosiglitazone. * *p* < 0.05, ** *p* < 0.01, *** *p* < 0.001. Values represent the means ± SEM of three independent experiments.

As a classic ligand-regulated nuclear receptor, the activity of FXR is regulated by the recruitment and release of co-regulators, including coactivators such as steroid receptor coactivators (SRCs), and corepressors such as nuclear corepressor (NCoR) [9]. To search for novel small FXR modulators, we performed high-throughput chemical screening with a natural product library (~2700 natural products) using a previously published commercial AlphaScreen assay (Figure 1B) that validated the efficacy of candidates in regulating the interaction affinity of FXR ligand-binding domain (FXR-LBD) protein with co-regulator binding motif peptides [33]. 

Fortunately, 2-oxokolavenol (CAS 130395-82-3) (Figure 1A), a natural product derived from *Aglaia spectabilis* with a chemical structure distinct from reported FXR agonists such as GW4064, OCA, or CDCA (Figure S1 in Supplementary Materials), potently induced FXR to recruit multiple coactivator binding motifs, including SRC1-2 and SRC2-3, but to a lesser extent than that of the reported FXR full agonist OCA in the AlphaScreen assay (Figure 1C). Additionally, the dose-response curve indicated that 2-oxokolavenol could induce FXR to recruit the SRC2-3 binding motif in a dose-dependent manner with an EC_50_ of approximately 3.7 μM in the AlphaScreen assay (Figure 1D), suggesting that 2-oxokolavenol is a novel FXR agonist. Generally, the binding of agonists to FXR will induces FXR to recruit coactivators and release corepressors. In addition to coactivator SRCs, we also performed the AlphaScreen assay to identify whether FXR regulates the corepressor NCoR2 motif in response to 2-oxokolavenol. The results demonstrated that 2-oxokolavenol could induce FXR-LBD to release the NCoR2 motif (Figure 1E), providing more powerful evidence that 2-oxokolavenol is an FXR agonist. Because the binding of agonists into ligand binding pockets will regulate the transcriptional activity of nuclear receptors, FXR can bind to the ecdysone response element (EcRE) and initiate the transcription of downstream genes. Cloning the luciferase gene downstream of EcRE to gain the reporter system (Figure 1F), we performed a cell-based dual-luciferase reporter assay to further confirm the agonist properties of 2-oxokolavenol. The results revealed that 2-oxokolavenol activated the transcriptional activity of FXR in a dose-dependent manner, with an EC_50_ of approximately 6.9 μM (Figure 1G). We also performed a dual-luciferase reporter assay to verify the selectivity of 2-oxokolavenol against other nuclear receptors. The results showed that 2-oxokolavenol selectively activated FXR without affecting other nuclear receptors tested, such as the peroxisome proliferator-activated receptor families (α, β and γ), pregnane X receptor (PXR), and the retinoid-related orphan receptor families (α, β and γ) (Figure 1H). These results suggested that the natural product 2-oxokolavenol, with an interesting chemical structure, is a novel and selective FXR agonist.

### 2.2. 2-Oxokolavenol Acts as an Agonist through Direct Interaction with FXR

**Figure 2:**
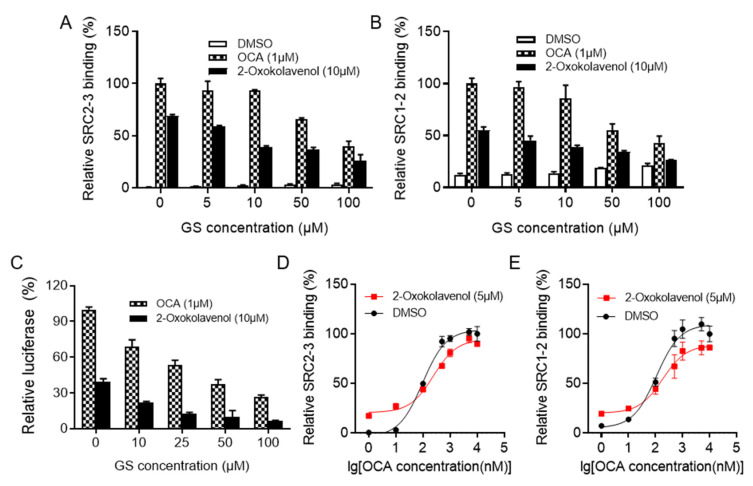
2-Oxokolavenol activities as an agonist through direct interaction with FXR. Guggulsterone E&Z (GS) is the first reported small molecule with antagonistic activity on FXR. (**A**,**B**) Gradient GS treatment blocked SRC2-3 (**A**) or SRC1-2 (**B**) binding motif recruitment induced by 2-oxokolavenol (10 μM) or OCA (1 μM) in the AlphaScreen assay. (**C**) Gradient GS treatment weakened the FXR transcriptional activity induced by 2-oxokolavenol (10 μM) or OCA (1 μM) in the cell-based dual-luciferase assay. (**D**,**E**) Dose response curves of OCA in inducing FXR-LBD to recruit SRC2-3 (**D**) or SRC1-2 (**E**) binding motifs with or without 5 μM 2-oxokolavenol in the AlphaScreen assay. Values represent the means ± SEM of three independent experiments.

Guggulsterone E&Z (GS) was the first reported small molecule with antagonistic activity against FXR [34]. In the AlphaScreen assay, 2-oxokolavenol potently induced FXR to recruit coactivators SRC2-3 and SRC1-2, both of which could be intercepted by gradient GS treatment (Figure 2A,B). In the cell-based dual-luciferase reporter assay, gradient GS also consistently inhibited the FXR transcriptional activity induced by 2-oxokolavenol (Figure 2C). OCA is a full FXR agonist with a strong ability to activate FXR, whereas 2-oxokolavenol had slightly lower activity against FXR compared to OCA. Thus, if 2-oxokolavenol bound to the same FXR ligand binding pocket as OCA, the presence of 2-oxokolavenol would inevitably intervene in the coactivator recruitment curve of FXR in response to OCA. Indeed, coactivator SRC (1-2 and 2-3) recruitment of FXR in response to OCA was more sensitive at low OCA concentrations in the AlphaScreen assay since there were sufficient FXR molecules for both OCA and FA binding, thus exerting positive collaborative effects in activating FXR. However, SRC (1-2 and 2-3) recruitment was less sensitive at high OCA concentrations since OCA and 2-oxokolavenol competed for binding to insufficient FXR molecules (Figure 2D,E). Given that 2-oxokolavenol could compete with both FXR antagonist GS and FXR agonist OCA to bind with FXR, these data reaffirmed that natural product 2-oxokolavenol acts as a novel FXR modulator.

### 2.3. Molecular Docking of 2-Oxokolavenol/FXR-LBD Complex and Functional Correlation with FXR Interactions

**Figure 3:**
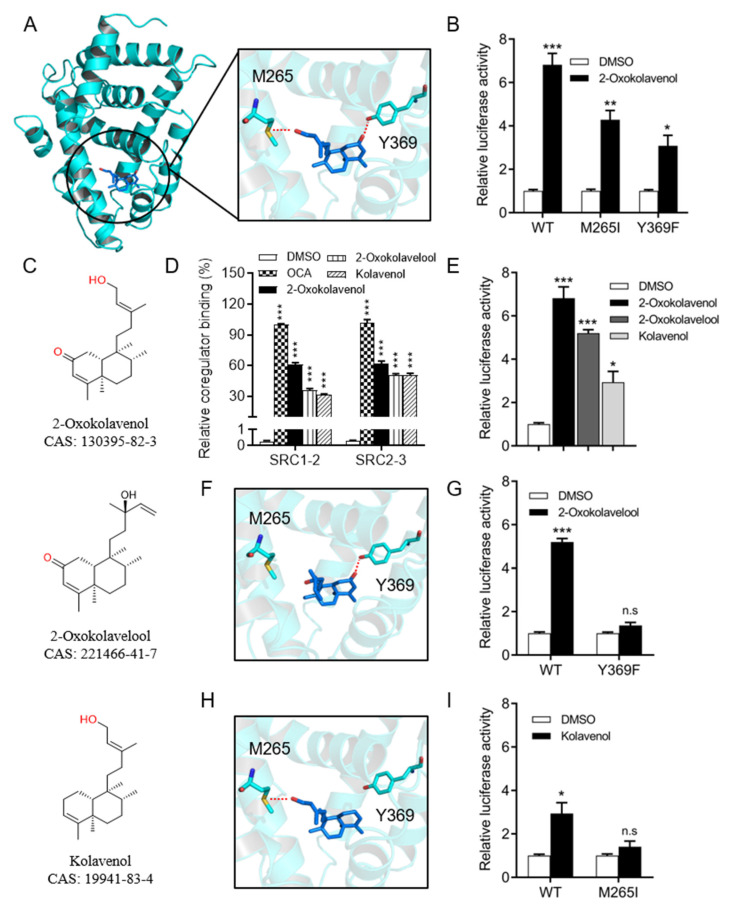
Molecular docking of 2-oxokolavenol/FXR-LBD complex and functional correlation with FXR interactions. (**A**) The molecular docking structure of 2-oxokolavenol (blue sticks) in the FXR ligand-binding domain pocket (green cartoon) based on a reported OCA/FXR-LBD complex co-crystal structure (PDB ID, 1OSV) using MOE software. (**B**) In the cell-based dual-luciferase reporter assay, wild-type and M265I/Y369F FXR mutants’ transcriptional activity in response to 2-oxokolavenol. (**C**) Chemical structure of 2-oxokolavenol, 2-oxokolavelool, and kolavenol. (**D**) SRC1-2 and SRC2-3 coactivator motifs bound to FXR-LBD in response to 2-oxokolavenol, 2-oxokolavelool, kolavenol, or OCA in the AlphaScreen assay. (**E**) A dual-luciferase reporter assay induced FXR transcriptional activity by 2-oxokolavenol, 2-oxokolavelool, kolavenol, or OCA. (**F**) The molecular docking structure of 2-oxokolavelool/FXR-LBD complex. (**G**) In a cell-based dual-luciferase reporter assay, wild-type and Y369F FXR mutants’ transcriptional activity in response to 2-oxokolavelool. (**H**) The molecular docking structure of the kolavenol/FXR-LBD complex. (**I**) In a cell-based dual-luciferase reporter assay, wild-type and M265I FXR mutants’ transcriptional activity in response to 2-oxokolavelool. In (**A**, **F**, and **H)**, hydrogen bonds between compounds and key residues are shown with dashed red lines. In (**B**, **E**, **G,** and **I**), plasmids encoding indicated full-length FXR (wild-type, M265I or Y369F mutants) and the cognate EcRE reporters were co-transfected into HEK293T (from ATCC). Six hours later, cells were treated with DMSO or indicated compounds, the luciferase signal was tested using a commercial dual-luciferase kit after 24 h. *p* values were determined by one-way ANOVA with Tukey’s multiple comparison post hoc test, ns, no significance, * *p* < 0.05, ** *p* < 0.01, *** *p* < 0.001. Values represent the means ± SEM of three independent experiments.

To better understand the binding pattern of 2-oxokolavenol with FXR-LBD, molecular docking simulations were performed using the crystal structure of the published OCA/FXR-LBD complex (PDB ID, 1OSV [22]) as a model. 2-Oxokolavenol compatibly bound into the classic FXR-LBD pocket, anchored by two hydrogen bonds with the phenolic hydroxyl of Y369 and sulfhydryl of M265 on FXR (Figure 3A). To confirm the significance of FXR key residues on the binding affinity and activity of 2-oxokolavenol based on the molecular docking model, we designed the M265I and Y369F mutants of FXR to abolish the hydrogen bond interactions with the hydroxyl and carbonyl groups of 2-oxokolavenol, respectively. As expected, the cell-based dual-luciferase reporter assay results revealed that M265I and Y369F significantly decreased the FXR transcriptional activity induced by 2-oxokolavenol (Figure 3B). Along with 2-oxokolavenol, the natural products 2-oxokolavelool (CAS 221466-41-7) and kolavenol (CAS 19941-83-4) could also induce FXR to recruit coactivator SRCs (1-2 and 2-3) in the AlphaScreen assay (Figure 3C,D) and activate the transcriptional activity of FXR in the cell-based dual-luciferase reporter assay (Figure 3E), but both with a lower affinity than that of 2-oxokolavenol. The oxygen atoms of compounds are often critical for hydrogen bonding to their protein targets, so the absence or position change of an oxygen atom in small molecules may result in blocked hydrogen bond formation with their protein targets, possibly altering the target binding affinity. To explain the differences in the activity of the three above-mentioned compounds targeting FXR, in addition to the 2-oxokolavenol/FXR-LBD complex, we also performed molecular docking simulations for the 2-oxokolavelool/FXR-LBD and kolavenol/FXR-LBD complexes. Compared with 2-oxokolavenol, 2-oxokolavelool has a variation in the hydroxyl group position and loses a hydrogen bond with the sulfur atom of M265 on FXR (Figure 3F). Similarly, the lack of carbonyl group in kolavenol leads to the loss of a hydrogen bond with Y369 on FXR when compared with 2-oxokolavenol (Figure 3H). Under the guidance of molecular docking, we also designed FXR mutants to verify the roles of key residues of FXR on 2-oxokolavelool or kolavenol binding and the subsequent FXR activity. Indeed, the Y369F mutant completely abolished the FXR transcriptional activity induced by 2-oxokolavelool (Figure 3G) and the M265I mutant abolished the kolavenol-induced transcription of FXR (Figure 3I). Collectively, our docking and mutagenesis studies suggested that the binding and activation of FXR by 2-oxokolavenol was mediated through direct interaction with M265/Y369 residues in the classic ligand-binding pocket of FXR.

### 2.4. 2-Oxokolavenol Alleviates APAP-Induced Hepatocytes Damage in an FXR-Dependent Manner in Human Liver WRL68 Cells

**Figure 4:**
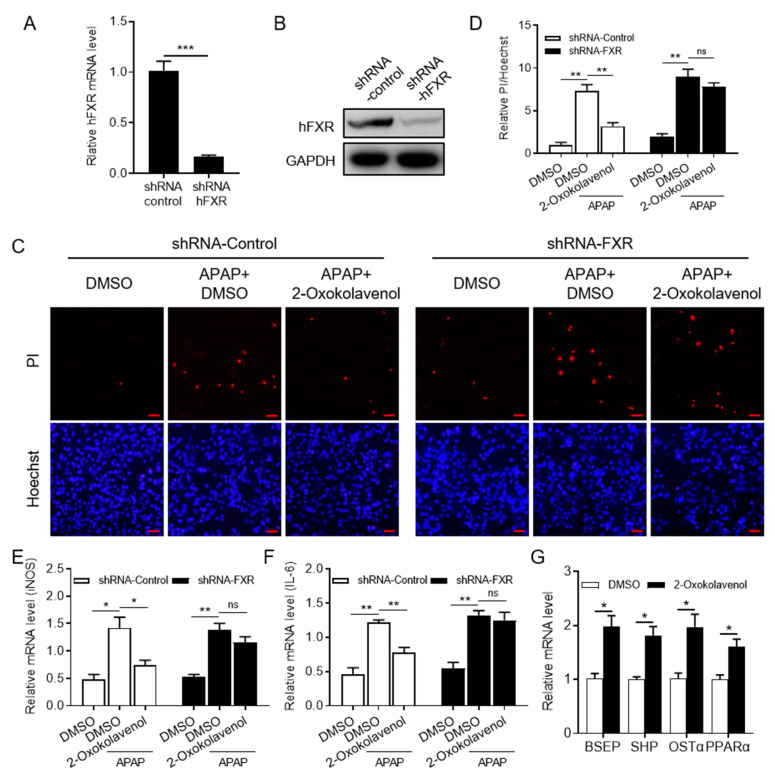
2-Oxokolavenol alleviated APAP-induced hepatocyte damage in a partial FXR-dependent manner. Human liver WRL68 cell line (from ATCC) with silenced FXR (shRNA-FXR WRL68) was established with lentivirus technology, and scrambled shRNA was used as control (shRNA-control RWRL68). (**A**,**B**) The efficacy of FXR silencing in shRNA-FXR WRL68 cells was identified by measuring FXR mRNA (**A**) and protein (**B**) levels. (**C**) The shRNA-control and shRNA-FXR WRL68 cells were treated with APAP for 6 h after pre-treatment with 2-oxokolavenol or DMSO for 18 h, then Hoechst33342 and propidium iodide (PI) stains were used. Hoechest33342 can stain all cells, but PI will only stain damaged cells due to inadequate membrane permeability. (**D**) Relative quantification of PI/Hoechst33342-positive cell ratios in fluorescent images from (**C**). (**E**) Relative iNOS mRNA levels. (**F**) Relative IL-6 mRNA levels. (**G**) Relative mRNA levels of FXR target genes in WRL68 cells. *p* values were determined by one-way ANOVA with Tukey’s multiple comparison post hoc test, ns, no significance, * *p* < 0.05, ** *p* < 0.01, *** *p* < 0.001. Values represent the means ± SEM of three independent experiments.

Given the critical regulation of FXR in hepatocyte metabolism and damage, we attempted to investigate the potential therapeutic efficacy of 2-oxokolavenol on APAP-induced hepatocyte damage as an FXR agonist. First, the WRL68 cell line (from ATCC) with the stable silencing of FXR (shRNA-FXR WRL68) was established by lentiviruses containing an shRNA-FXR construct, and the viruses containing a scrambled shRNA construct were used as a negative control (shRNA-control WRL68). The mRNA (Figure 4A) and protein expression levels (Figure 4B) of FXR in shRNA-FXR and shRNA-control WRL68 cells were measured to determine the effective knockdown of FXR. Then, shRNA-control WRL68 and shRNA-FXR WRL68 cells were used to verify whether 2-oxokolavenol could relieve APAP-induced hepatocyte damage in an FXR-dependent manner. Hoechst33342 and propidium iodide (PI) were used to distinguish healthy and damaged cells because PI is unable to penetrate intact cell membranes and stain healthy cells, but Hoechst33342 can stain both healthy and damaged cells. According to the Hoechst33342/PI double staining results, 2-oxokolavenol treatment significantly alleviated APAP-induced hepatocyte damage in shRNA-control WRL68 cells, but the efficacy was deprived in shRNA-FXR WRL68 cells (Figure 4C,D). Then, we tested the mRNA levels of inflammation-related biomarkers, such as inducible nitric oxide synthase (iNOS) and interleukin (IL)-6, to further confirm the FXR dependence of 2-oxokolavenol for reducing the APAP-induced inflammatory response in hepatocytes. Consistent with the Hoechst33342/PI double staining results, 2-oxokolavenol treatment restored the elevated iNOS and IL-6 mRNA levels induced by APAP treatment in the shRNA-control WRL68 cells but not in the shRNA-FXR WRL68 cells (Figure 4E,F). The binding of different ligands into the FXR pocket will induce FXR to regulate the expression of downstream target genes to exert various biological activities. Intriguingly, 2-oxokolavenol treatment increased the transcription of multiple FXR target genes, such as bile salt export pump (BSEP), small heterodimer partner (SHP), organic solute transporter subunit alpha (OSTα), and peroxisome proliferator-activated receptor alpha (PPARα), in WRL68 cells (Figure 4G). These results suggested that 2-oxokolavenol exerted its efficacy on APAP-induced hepatocyte damage by directly targeting and activating FXR.

## 3. Discussion

Considering the crucial roles of FXR in maintaining and reversing aberrant metabolism in the liver, FXR has been confirmed to be a promising drug target for chronic and acute liver disorders. However, OCA is the only approved drug that targets FXR to date, and several serious safety issues have emerged in multiple clinical trials, including jaundice, increased low-density lipoprotein cholesterol levels, reduced high-density lipoprotein cholesterol levels, and especially, dose-dependent severe itching, which have indisputably limited its further clinical use [10,11,28]. Therefore, the discovery of potent and selective FXR modulators with novel chemical scaffolds is an attractive strategy for drug discovery that may satisfy the clinical drug requirements for FXR-mediated liver diseases.

Herein, an AlphaScreen-based high-throughput chemical screening assay was established using an *in vitro* commercial kit to find novel FXR agonists from a commercial compound library of natural products with diverse skeletons, and the natural product 2-oxokolavenol was initially identified as a selective and bioactive FXR agonist. In detail, 2-oxokolavenol could not only induce FXR to recruit coactivator binding motifs SRC1-2 and SRC2-3, but it could also induce FXR to release corepressor binding motif NCoR2 in the AlphaScreen assay, which confirmed the characteristics of 2-oxokolavenol as an FXR agonist. The results of the cell-based dual-luciferase reporter tests indicated that 2-oxokolavenol can selectively induce the transcriptional activity of FXR without affecting multiple other nuclear receptors, such as PXR, PPARs, and RORs, which again verified 2-oxokolavenol as an FXR agonist. In order to understand the molecular mechanism of 2-oxokolavenol binding FXR, we performed a molecular docking simulation between 2-oxokolavenol and the reported FXR-LBD x-ray structure, which demonstrated that 2-oxokolavenol forms two hydrogen bonds with M265 and Y369 of human FXR-LBD to compatibly fit into the ligand binding pocket of FXR. Finally, an APAP-induced hepatocyte damage cell model was chosen to determine the pharmacological potential of 2-oxokolavenol as a novel FXR agonist. The results indicated that 2-oxokolavenol treatment can alleviate APAP-induced hepatocyte damage in an FXR-dependent manner in WRL68 cells. In summary, these results provide suficient evidence for future drug candidate design based on the natural product 2-oxokolavenol by directly targeting FXR.

With a wide range of active and structural components, natural products have always been one of the main treasure troves for drug molecules. Many approved drugs are natural products or their derivatives [31,35]. Isolated from *Amoora stellato-squamosa,* 2-oxokolavenol does not have any previously reported biological activities. Moreover, *Amoora stellato-squamosa* does not have any proven medicinal properties to date. Thus, it is of great scientific significance to study whether 2-oxokolavenol has potential pharmacologic targets and explore their underlying mechanisms of action. Herein, FXR was identified as a direct target of 2-oxokolavenol, and more importantly, 2-oxokolavenol demonstrated pharmacologic efficacy against hepatocyte disorders by activating FXR. On the one hand, this discovery confirms a direct pharmacologic target of the natural product 2-oxokolavenol. On the other hand, the study findings support the potential medicinal value of plants capable of producing 2-oxokolavenol.

The supply of natural products is limited and variable. Directly isolating a large amount of natural products from plants in order to support biological exploration and potential drug candidate development will inevitably cause irreversible damage to nature. To obtain enough 2-oxokolavenol to enable follow-up biological studies in vivo, efficient chemical synthesis is a suitable solution. In addition, compared with OCA, 2-oxokolavenol still has great room for improvement in the activation of FXR (Figure 1D,F). Thus, modification of 2-oxokolavenol to a derivative with better FXR affinity is an option for future study. Meanwhile, in order to more efficiently and rationally design and synthesize derivatives, we hope to obtain the structure of the FXR-LBD/2-oxokolavenol complex to guide the direction of synthetic design. Unfortunately, although many attempts have been made, we have not yet succeeded. All in all, based on the crucial roles of FXR in regulating liver homeostasis and the reported evidence in this study, the discovery and biological validation of 2-oxokolavenol as a novel FXR agonist imply its significant potential for drug development to meet clinical needs in the future. We will continue to try to obtain the structure of the FXR-LBD/2-oxokolavenol complex, carry out the total synthesis, and optimize derivatives of 2-oxokolavenol in future work. 

Collectively, the approval of OCA by the FDA for PBC treatment in the clinic confirms the reliability of FXR as a drug target for liver damage. The natural product 2-oxokolavenol has been demonstrated here to be a novel and selective FXR agonist with the ability to relieve hepatocyte damage. Thus, it is conceivable that the modification of 2-oxokolavenol to create additional derivatives with optimized chemical structures may provide an effective mean for the development of new drug candidates for the treatment of FXR-mediated liver diseases in the future. 

## 4. Materials and Methods

### 4.1. Protein Expression and Purification

The human 6His-FXR-LBD (residues 243-472aa) was cloned on the prokaryotic expression vector pET28a(+) (Beijing Zoman Biotechnology Co., Ltd.). BL21 (DE3) *E. coli* cells (Beijing Zoman Biotechnology Co., Ltd.) with transfected pET28a(+)-6His-FXR-LBD plasmids were cultivated in lysogeny broth at 37 °C to an OD600 of approximately 1.0. Then, 0.2 mM isopropyl 1-thio-b-D-galactopyranoside was added after the temperature was stabilized at 16 °C. *E. coli* cells were harvested and sonicated in 200 mL extraction buffer (20 mM Tris, pH 7.4, 300 mM NaCl, 5% glycerol, and 25 mM imidazole) per 6 L cells. The cell lysate was centrifuged at 15,000 rpm for 30 min, and the supernatant was loaded on a 5 mL HisTrap prepacked column (GE Healthcare) after filtration. The column was washed with washing buffer (20 mM Tris, pH 7.4, 150 mM NaCl, 5% glycerol, and 25 mM imidazole) and the target protein was eluted with a gradient imidazole (25–500 mM). The FXR-LBD protein was further purified with a HiLoadTM 16/600 molecular sieve (GE Healthcare). 

### 4.2. AlphaScreen Co-Regulators Binding Assay

The binding of multiple co-regulator motif peptides to FXR-LBD protein in response to compounds was determined by AlphaScreen assay (the schematic diagram is shown in Figure 1B) using a hexahistidine detection kit (Perkin-Elmer, code 6760619R). For screening (natural product library from BioBioPha company, China. http://www.biobiopha.com/ (accessed on 14 November December 2022)), small molecules were transferred into a 384-well OptiPlate by Echo520 (Beckman Coulter, Inc. California, USA), the mixture containing FXR-LBD and biotin-SRC2-3 was transferred into the OptiPlate wells by a multidrop microplate dispenser (Thermo Fisher, USA), and AlphaScreen signaling was measured using an Envision reader (Perkin-Elmer, USA) after incubation for 1 h at room temperature. The assay was performed using approximately 20~40 nM 6His-FXR-LBD protein and approximately 20 nM biotinylated co-regulator motif peptides in the presence of 2~5 μg/mL donor beads (streptavidin-coated) and acceptor beads (nickel-coated) in a buffer containing 50 mM MOPS, 50 mM NaF, 0.05 mM CHAPS, and approximately 0.1 mg/mL BSA, pH 7.4. The following biotinylated co-regulator motif peptides were used: 

SRC1-2, biotin-SPSSHSSLTERHKILHRLLQEGSP

SRC2-3, biotin-QEPVSPKKKENALLRYLLDKDDTKD 

NCoR-2, biotin-GHSFADPASNLGLEDIIRKALMGSF

### 4.3. Cell-Based Lluciferase Reporter Assay

A cell-based luciferase reporter assay was used to evaluate the ability of the tested compounds to induce FXR transcriptional activity. In brief, the luciferase gene was cloned downstream of the cognate response element of nuclear receptors, the binding of nuclear receptors to their cognate response element induced the expression of luciferase, and the luciferase activity was measured using the dual-luciferase kit (Promega) (the schematic diagram of luciferase reporter assay is shown in Figure 1F).

HEK293T (from ATCC) was cultured in Dulbecco’s modified Eagle’s medium containing 10% fetal bovine serum at 37 °C under 5% CO_2_. All plasmids were isolated using a commercial endotoxin-free plasmid kit. All mentioned mutant FXR plasmids were created using the Quick-Change site-directed mutagenesis kit, and the sequences of primer pairs for cloning the human FXR full-length mutant plasmids are summarized in Supplementary Table S1. HK293T cells were seeded into 96-well plates before transfection, then co-transfected by PEI with plasmids encoding the full-length nuclear receptors and their cognate luciferase reporters as follows: human FXRα and EcRE-luciferase, human peroxisome proliferator activated receptors (α,β,γ) with PPRE-luciferase, human PXR and constitutive androstane receptor with PBRE-luc reporter, and human retinoic acid-related receptors (α,β,γ) with the Pcp2/RORE-luciferase. Specific and tested candidates were added 6 h after transfection. Luciferase activity was measured 24 h later using the dual-luciferase kit (Promega) with an Envision reader. The luciferase activities were normalized to renilla activity, using the co-transfected plasmid as an internal control.

### 4.4. Docking between FXR-LBD and Compounds

The FXR-LBD/2-oxokolavenol, FXR-LBD/2-oxokolavelool, and FXR-LBD/kolavenol molecular docking simulations were performed based on the reported structure of the FXR-LBD/OCA complex (PDB ID, 1OSV). One monomer structure of the FXR-LBD/OCA complex dimer was isolated and the OCA was removed with MOE software. The three compounds mentioned above were docked into the ligand-binding pocket of FXR-LBD with standard parameters. Pymol was used to output the final pictures. 

### 4.5. Establishment of WRL68 Cell Line with the Stable Silence of FXR

WRL68 cells (from ATCC) were cultured in Dulbecco’s modified Eagle’s medium containing 10% fetal bovine serum at 37 °C under 5% CO_2_. Lentivirus technology was used to stably silence NR1H4 (coding for the FXR protein) in the human WRL68 cell line. Packaging plasmids (VSV-G, pRSV rev, and pMDLg/pRRE) were co-transfected with the human FXR-shRNA or negative-shRNA plasmid (scramble) into HEK293T cells to produce sufficient targeted retrovirus. The supernatants were collected 48 and 72 h after transfection, mixed, centrifuged, and used to infect WRL68 cells. Puromycin (2 μg/mL) was added to the medium to exclude the uninfected cells after infection for 48 h. Finally, western blot (anti-FXR antibody, ABclonal, #A8320) and qPCR were employed to verify the efficacy of FXR knockdown. The shRNA sequence of the human FXR gene is as follows: CCGGCCACTTCTTGATGTGCTACAACTCGAGTTGTAGCACATCAAGAAGTGGTTTTT. 

### 4.6. APAP-Induced Hepatocytes Damage Cell Model

WRL68 cells (from ATCC) were cultured in culture dishes with Dulbecco’s modified Eagle’s medium containing 10% fetal bovine serum at 37 °C under 5% CO_2._ APAP (acetaminophen) was added to the medium after pre-treatment with 2-oxokolavenol or DMSO for 18 h. After a total of 24 h, the WRL68 cells were stained with Hoechst33342 and propidium iodide (PI) for 15 min. Images were collected using a fluorescence microscope (OLYMPUS, IX73).

### 4.7. RNA Isolation and qPCR

RNA isolation (Code R6934, OMEGA Bio-Tek, GA), first-strand synthesis of cDNA (Code 11141ES10, Yeasen, China), and reverse transcription using SYBR mixture (Code 11201es08, Yeasen, China) for qPCR (CFX96, Bio-Rad) to verify mRNA levels were performed according to the manufacturers’ standard instructions. Relative mRNA expression was normalized to that of GAPDH. The sequences of the primer pairs are listed in Supplementary Table S2.

### 4.8. Statistical Analysis

Values in this study were expressed as mean ± standard error of the mean (SEM). The student t-test was used for comparisons with only two groups. Analysis of variance (ANOVA) with Tukey’s post-hoc test was used to compare values among different experimental groups (one-way ANOVA for comparisons between groups; two-way ANOVA for comparisons of changes between other groups with various interventions).

## Data Availability

All data are listed in tables or presented in figures in the main text or Supplementary Materials.

## Supplementary Materials

The following supporting information can be downloaded at: https://www.mdpi.com/article/10.3390/molecules27248968/s1. Figure S1: Chemical structures of 2-Oxokolavenol and other well-known FXR ligands. GW4064 is a synthetic full agonist for FXR, CDCA is a physiological agonist, and OCA is a semisynthetic CDCA derivative with improved activity; Figure S2: Full scans of western blot for Figure 4B; Table S1: The sequences of primer pairs for cloning human FXR full length mutant plasmids; Table S2: The sequences of primer pairs for determining mRNA level through qPCR in this study.

## Sample Availability

Samples of the 2-oxokolavenol (CAS 130395-82-3), 2-oxokolavelool (CAS 221466-41-7) and kolavenol (CAS 19941-83-4) were purchased from company (BioBioPha, China).
