# Supplementary material for: Natural Product 2-Oxokolavenol Is a Novel FXR Agonist
Source: Molecules. 2022 Dec 16;27(24):8968. doi: 10.3390/molecules27248968 (PMC9786051; doi:10.3390/molecules27248968)
Supplement: Supplementary file 1 [file molecules-27-08968-s001.zip › molecules-2067460-SI.pdf]

Supplementary Information

Natural product 2-Oxokolavenol is a novel FXR agonist

Fusheng Guo [1,2,†], Yihui Gao [1,†], Xiaobao Li [3,*], Xiaoguang Lei [1,2,*]

[1] Beijing National Laboratory for Molecular Sciences, Key Laboratory of Bioorganic Chemistry and Molecular Engineering of Ministry of Education, Department of Chemical Biology, College of Chemistry and Molecular Engineering, Synthetic and Functional Biomolecules Center, Peking University, Beijing 100871, China
[2] Peking-Tsinghua Center for Life Science, Academy for Advanced Interdisciplinary Studies, Peking University, Beijing 100871, China
[3] Key Laboratory of Tropical Medicinal Resource Chemistry of Ministry of Education &Key Laboratory of Tropical Medicinal Plant Chemistry of Hainan Province, College of Chemistry and Chemical Engineering, Hainan Normal University, Haikou 571158, China
* Correspondence: lixiaobao0797@163.com (X.B.L.). or xglei@pku.edu.cn (X.G.L.).
† These authors contributed equally to this work.

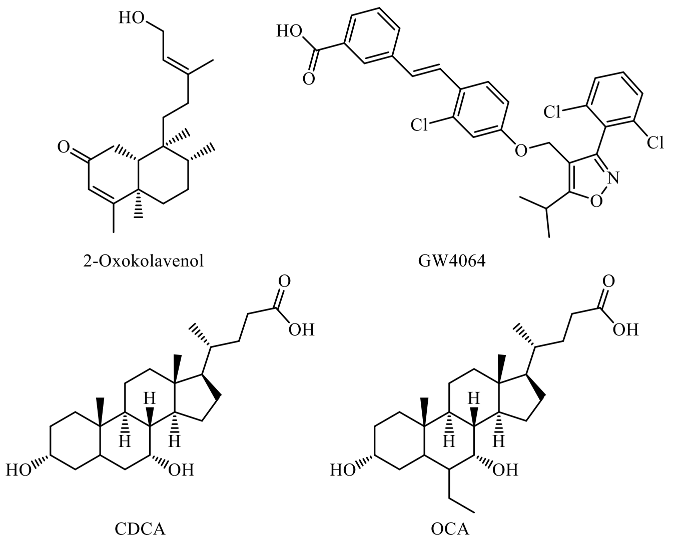

Supplementary Figure S1. Chemical structures of 2-Oxokolavenol and other well-known FXR ligands. GW4064 is a synthetic full agonist for FXR, CDCA is a physiological agonist, and OCA is a semisynthetic CDCA derivative with improved activity.

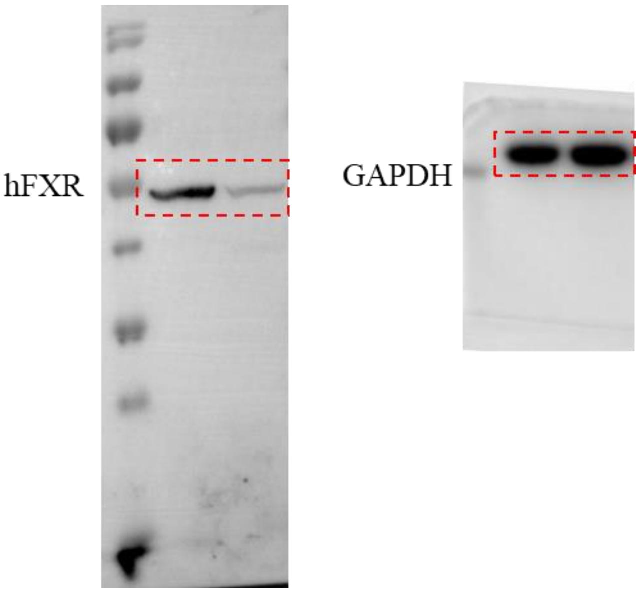

Supplementary Figure S2. Full scans of western blot for figure 4B

Supplementary table S1. The sequences of primer pairs for cloning human FXR full length mutant plasmids.

Primer pairs (5'-3') for mutations (hFXR)		
M265I	Forward	GGATTCATATAACAAACAGAGGATCCCTCAGGAAATAACAAATAAAAT
	Reverse	ATTTTATTTGTTATTTCCTGAGGGATCCTCTGTTTGTTATATGAATCC
Y369F	Forward	TATAACACCTATGTTTAGTTTTTTAAAAGTATTGGGGAACTGAAAATG
	Reverse	CATTTTCAGTTCCCCAATACTTTTAAAAAAACTAAACATAGGTGTTATA

Supplementary table S2. The sequences of primer pairs for determining mRNA level through qPCR in this study.

Gene name (human)	Forward primer (5'-3')	Reverse primer (5'-3')
GAPDH	GAAGGTGAAGGTCGGAGT	GATGGCAACAATATCCACTT
IL-6	AGAGTAGTGAGGAACAAGCCAGAG	GCTACATTTGCCGAAGAGCC
iNOS	CCGGCAGACTGGATTTGG	TTGGGTCTCCGCTTCTCG
BSEP	GCCGCAGCTCGTCAGATAC	GAATTGCAGTCAAACCACCCTAT
Shp	GTGCCCAGCATACTCAAGAAG	TGGGGTCTGTCTGGCAGTT
OSTα	AGCAGAACATGGGAGCCAAA	TAGGGAGGCGAACAAGCAAT
PPARα	TTCGCAATCCATCGGCGAG	CCACAGGATAAGTCACCGAGG
